# Proton pump inhibitors-induced thrombocytopenia: A systematic literature analysis of case reports

**DOI:** 10.1515/med-2025-1297

**Published:** 2025-10-29

**Authors:** Xiaofei Yue, Hongjiao Tian

**Affiliations:** Rehabilitation Pharmacy Center, Beijing Rehabilitation Hospital, Capital Medical University, Beijing, 100144, PR China

**Keywords:** proton pump inhibitors, thrombocytopenia, adverse drug reaction, case

## Abstract

**Objective:**

Thrombocytopenia induced by proton pump inhibitors (PPIs) is a relatively uncommon adverse effect of this widely prescribed class of drugs. The objective of this study is to investigate the clinical features of PPIs-induced thrombocytopenia based on published case reports.

**Methods:**

We searched the PubMed, Web of Science, Scopus, China National Knowledge Infrastructure, Wanfang Data, and Chinese VIP databases from inception to August 2024 to identify reported cases of thrombocytopenia associated with PPIs use. Clinical data such as patient demographics, drug use information, adverse reactions, and outcomes were extracted and analyzed.

**Results:**

Overall, 16 publications describing 18 cases (12 males and 6 females) were included in this study, comprising a neonate and 17 adults with a median age of 62 years (range 23–98). The PPIs associated with thrombocytopenia included pantoprazole (11 cases), lansoprazole (4 cases), omeprazole (2 cases), and esomeprazole (1 case). The median time to symptoms onset was 3 days (range 2–7) after the initiation of PPI therapy. After discontinuation of PPIs and interventions such as platelet transfusion, 11 patients achieved recovery, and the remaining 7 patients experienced symptomatic improvement.

**Conclusion:**

Thrombocytopenia induced by PPIs appears to be a rare adverse event. Clinicians should enhance their awareness when evaluating potential cases of thrombocytopenia. Once PPIs are suspected, immediate discontinuation of the drugs and initiation of appropriate treatments are recommended.

## 1 Introduction

Proton pump inhibitors (PPIs) represent an important class of drugs that are widely prescribed for treatment of gastric acid-related disorders such as gastroesophageal reflux disease (GERD), gastritis, esophagitis, Barrett’s esophagus, Zollinger-Ellison syndrome, peptic ulcer disease, nonsteroidal anti-inflammatory drug-associated ulcers, and *Helicobacter pylori* (*H. pylori*) infection [1]. PPIs exert their inhibitory effect on gastric acid secretion by selectively and irreversibly blocking the H^+^/K^+^-ATPase enzyme system which is located in the gastric parietal cells [2,3].

Since the US Food and Drug Administration approved omeprazole as the first PPI in the late 1980s, other drugs in the same class – including pantoprazole, lansoprazole, esomeprazole, rabeprazole, and ilaprazole – have subsequently been marketed [4]. PPIs are generally considered as safe and well tolerated with minimal side effects, especially when used for short periods. During short-term treatment, patients may experience nonspecific symptoms such as headache, constipation, diarrhea, nausea, and vomiting [5], whereas prolonged use has been associated with other adverse effects, including infections, bone fractures, and renal damage [6]. However, with the growing prevalence of PPIs, a range of rare adverse drug reactions (ADRs) are increasingly emerging, such as thrombocytopenia.

Thrombocytopenia is a disorder characterized by a decreased number of platelets in peripheral blood, with multiple etiologies encompassing both congenital and acquired causes [7]. Drug-induced thrombocytopenia (DIT) is a specific yet often overlooked factor among these diverse causes. More than 300 drugs have been implicated with DIT. Among which, the most frequently reported with definite or probable causal relation to thrombocytopenia were quinine, quinidine, vancomycin, penicillin, oxaliplatin, and so on [8]. It is estimated that up to 25% of critically ill patients are at risk of developing DIT and the overall worldwide incidence can be as high as 10 cases per million people per year [7].

In recent years, the number of literature reports on PPIs-related thrombocytopenia has been increasing. Nevertheless, few systematic studies have investigated this potential risk or characterized patients with PPI use who experienced thrombocytopenia. Therefore, this study conducted a comprehensive analysis by searching relevant published case reports with the aim to provide a reference for clinical practice.

## 2 Materials and methods

### 2.1 Search strategy

This study was reported in accordance with Preferred Reporting Items for Systematic Reviews and Meta-Analyses (PRISMA) checklist (Table S1). A comprehensive search was conducted in PubMed, Web of Science, Scopus, China National Knowledge Infrastructure (CNKI), Wanfang Data, and Chinese VIP databases to identify case reports and case series of PPIs-induced thrombocytopenia published in Chinese or English from inception to August 2024 using the search terms: proton pump inhibitors, omeprazole, esomeprazole, pantoprazole, lansoprazole, rabeprazole, ilaprazole, thrombocytopenia, and platelet count. The bibliographies of all included publications and other publications of interest were manually searched for additional relevant studies. A detailed search strategy applied to these databases is provided in Table S2.

### 2.2 Inclusion and exclusion criteria

Studies were included or excluded in accordance with the predefined inclusion and exclusion criteria.

Inclusion criteria were as follows: original case reports or case series reporting thrombocytopenia due to the use of PPIs and detailed information was given.

Exclusion criteria were as follows: reviews, clinical trials, experimental studies, conference abstracts, full-text unavailable, and cases with incomplete or missing data.

### 2.3 Article selection

Two authors independently screened the titles and abstracts generated by the search strategy to assess eligibility for inclusion. Afterward, the full-text of eligible articles were retrieved and reviewed for final decision according to the selection criteria described above. All discrepancies were resolved by discussion between reviewers.

### 2.4 Data extraction

For each case, data regarding demographic and clinical characteristic were extracted and analyzed. Extracted information included, but was not limited to, basic details, PPIs involved, laboratory examinations, symptoms, treatments, clinical outcomes, and rechallenge. Naranjo scale was calculated to assess the probability of causality [9]. Severity of thrombocytopenia was graded according to the following classification: mild (100–149 × 10^9^/L), moderate (50–99 × 10^9^/L), and severe (<50 × 10^9^/L) [10].

### 2.5 Quality assessment

The Joanna Briggs Institute critical appraisal checklist was used to evaluate the quality of the selected case reports [11]. The intra-class correlation coefficient (ICC) statistic was calculated to evaluate the inter-rater agreement.

## 3 Results

**Figure 1:**
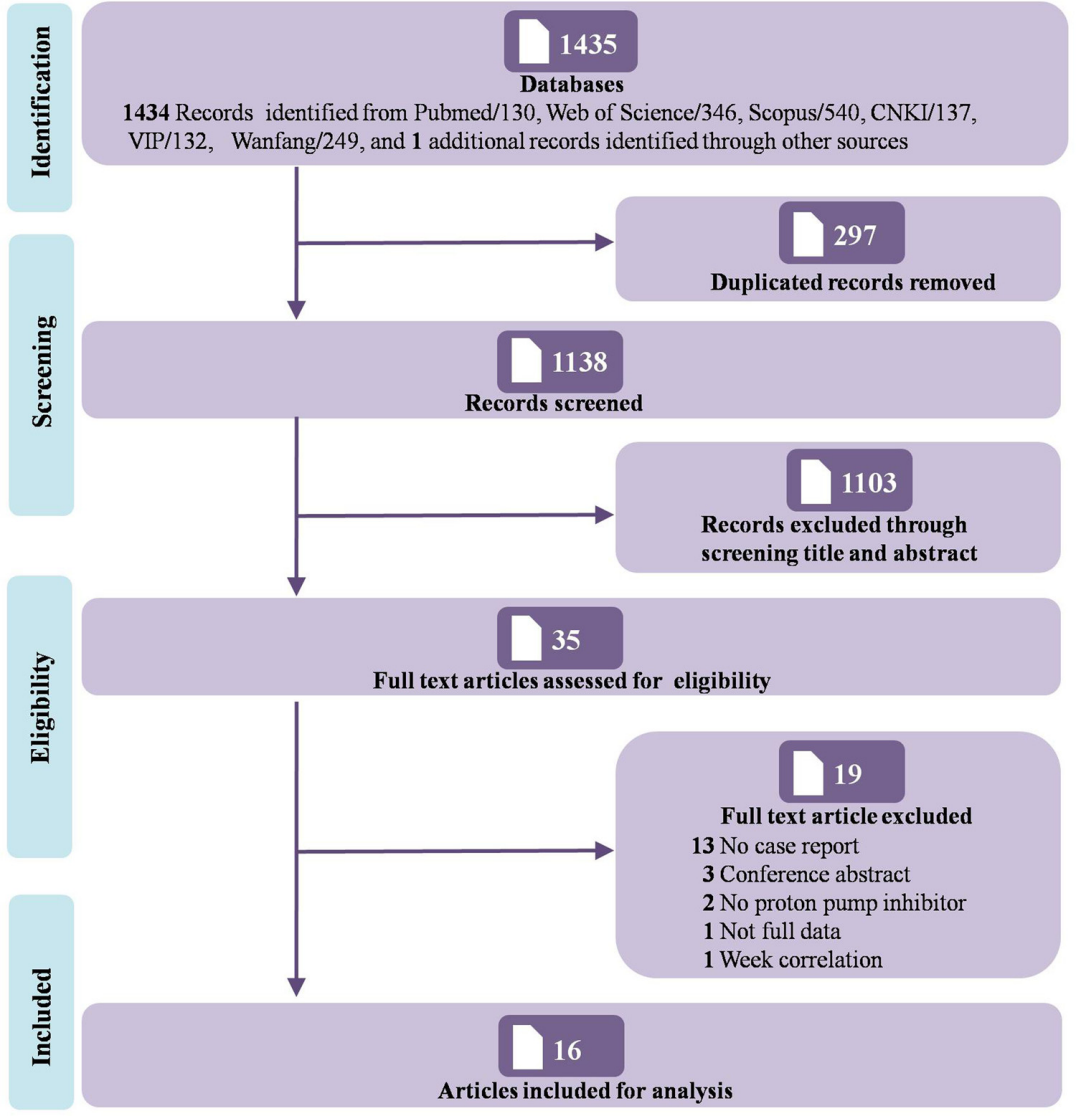
PRISMA flow diagram.

The detailed article selection process is illustrated in the PRISMA diagram (Figure 1). We finally included 16 articles in this study, representing a total of 18 independent patients. The cases were derived from 13 case reports [12–19,21,23,24,26,27] and 3 letters [20,22,25]. Data extracted from each individual case are summarized in Table 1, and the clinical characteristics of patients are presented in Table 2. All included studies underwent critical appraisal (Table S3). To summarize, 44.44% (8/18) of the included case reports were considered as being of high-quality evidence, 50.0% (9/18) as moderate-quality, and 5.56% (1/18) as low-quality. The ICC value between the two reviewers was 0.914, indicating very good agreement.

**Table 1:** Published reports of PPIs-associated thrombocytopenia

Case	Region	Age/sex	PPI	Dose	Baseline platelet count (×10^9^/L)	Time of onset (days)	Platelet nadir (×10^9^/L)	Bleeding sign or symptom	Treatment	Outcome	Outcome time (days)	Rechallenge	Naranjo scale	Previous exposure to PPI
Mukherjee et al. [12]	USA	35/F	Esomeprazole	IV, bid	116	2	12	No	Discontinued	Improved	2	No	Probable^a^	Yes
Liu and Han [13]	China	76/M	Omeprazole	20 mg, PO, qd	NR	4	48	Purple spot	Discontinued and dexamethasone therapy	Improved	7	No	Possible^a^	NR
Liu and Han [13]	China	23/M	Omeprazole	20 mg, PO, qd	NR	2	28	Petechiae, ecchymosis	Discontinued	Recovered	10	No	Possible^a^	NR
Saad and Mitwally [14]	Qatar	50/M	Lansoprazole	30 mg, NG, qd	315	5	57	No	Discontinued and changed to ranitidine	Recovered	3	Yes (recurrence)	Probable	Yes
Zlabek and Anderson [15]	USA	85/M	Lansoprazole	60 mg, PO, bid	160	2	36	No	Discontinued and changed to ranitidine	Recovered	7	No	Possible	No
Deng et al. [16]	China	82/F	Lansoprazole	30 mg, IV, qd	108	3	64	No	Discontinued	Improved	4	Yes (recurrence)	Probable^a^	NR
Zheng and Wen [17]	China	68/M	Lansoprazole	30 mg, IV, bid	94	7	27	No	Discontinued, rhIL-11 injection, and platelet transfusions	Recovered	5	Yes (recurrence)	Probable^a^	NR
Yu et al. [18]	China	85/M	Pantoprazole	80 mg, IV, bid	NR	3	83	No	Discontinued	Recovered	8	No	Probable	Yes
Phan et al. [19]	USA	66/F	Pantoprazole	40 mg, IV, bid	193	7	<20	No	Discontinued, changed to famotidine and platelet transfusions	Recovered	9	No	Probable^a^	NR
Korkmaz et al. [20]	Turkey	98/M	Pantoprazole	80 mg, IV, then infusion at 8 mg/h	160	2	54	No	Discontinued	Recovered	4	No	Probable^a^	No
Widyati et al. [21]	Indonesia	51/M	Pantoprazole	40 mg, IV, qd	42	2	18	Oral mucosa bleeding	Discontinued	Improved	2	No	Probable	Yes
Miller et al. [22]	USA	9 d/F	Pantoprazole	1 mg/kg/day increased to 1 mg/kg/12 h	241	7	37	No	Discontinued, changed to omeprazole and platelet transfusions	Recovered	4	No	Possible	Yes
Watson et al. [23]	USA	62/F	Pantoprazole	40 mg, PO, qd	340	6	87	No	Discontinued	Recovered	3	No	Probable^a^	No
Watson et al. [23]	USA	42/M	Pantoprazole	40 mg, PO, qd, then changed to IV	244	5	75	No	Discontinued and changed to lansoprazole	Improved	6	No	Probable^a^	Yes
Kallam et al. [24]	USA	50/M	Pantoprazole	40 mg, PO, qd	177	2	47	No	Discontinued and changed to famotidine	Recovered	7	No	Probable^a^	Yes
Tas [25]	Turkey	45/M	Pantoprazole	80 mg, IV, then infusion 8 mg/h	350	3	70	No	Discontinued, after improved changed to rabeprazole	Improved	4	No	Probable^a^	NR
Sahad et al. [26]	India	55/M	Pantoprazole	80 mg, IV, qd	177	2	57	No	Discontinued and changed to omeprazole	Improved	NR	No	Probable	NR
Xie et al. [27]	China	77/F	Pantoprazole	80 mg, IV, bid	208	3	39	No	Discontinued and changed to famotidine	Recovered	5	No	Probable^a^	NR

NR: not reported; PO: per os; IV: intravenous; ^a^Naranjo scale were calculated by authors from available information.

**Table 2:** Characteristic of the 18 included patients

Variable		No. of patients (%)
Gender	Male	12 (66.67)
	Female	6 (33.33)
Age	Newborn	1 (5.56)
	20–49	4 (22.22)
	50–79	9 (50)
	80–99	4 (22.22)
Indication for PPIs	Stress ulcer prophylaxis	5 (27.78)
	Gastrointestinal bleeding	4 (22.22)
	Abdominal pain	2 (11.11)
	Peptic ulcer	2 (11.11)
	Acid reflux	2 (11.11)
	GERD	1 (5.56)
	Nonspecific gastritis	1 (5.56)
	Not reported	1 (5.56)
Distribution of PPIs	Pantoprazole	11 (61.11)
	Lansoprazole	4 (22.22)
	Omeprazole	2 (11.11)
	Esomeprazole	1 (5.56)
Onset time	0–1 days	0
	2–3 days	11 (61.11)
	4–5 days	3 (16.67)
	6–7 days	4 (22.22)
Classification of thrombocytopenia	Mild (100–149 × 10^9^/L)	0
	Moderate (50–99 × 10^9^/L)	8 (44.44)
	Severe (<50 × 10^9^/L)	10 (55.56)
Treatment	Discontinuation	18 (100)
	Discontinuation with other treatment	4 (22.22)
Outcome	Improved	7 (38.89)
	Recovery	11 (61.11)

Out of these cases, 12 (66.67%) were males and 6 (33.33%) were females. The age of patients spanned from 23 to 98 years, with one 9-day-old newborn reported. The median age of adult patients was 62 years and most (13/18, 72.2%) were 50 years or older. Patients were from the USA (*n* = 7), China (*n* = 6), Turkey (*n* = 2), India (*n* = 1), Indonesia (*n* = 1), and Qatar (*n* = 1).

In these cases, thrombocytopenia was identified by the use of pantoprazole (*n* = 11), followed by lansoprazole (*n* = 4), omeprazole (*n* = 2), and esomeprazole (*n* = 1). Seven patients had documented prior exposure to PPIs, three of whom had a history of platelet counts dropping [21,23,24]. Indications for PPIs therapy were various. Stress ulcer prophylaxis (*n* = 5) and gastrointestinal bleeding (*n* = 4) were the most frequently reported. Others were abdominal pain, peptic ulcer, acid reflux, GERD, etc. The dose of a neonatal patient was calculated by body weight, and the initial dose was increased from 1 mg/kg/day to 1 mg/kg/12 h. For the other adults, the daily dose of PPIs was used at a standard dose (20–160 mg/day), with no overdose reported.

Thrombocytopenia can occur as early as 2 days after starting PPI therapy, and also up to 7 days. All cases had their reaction onset times documented and the median time was 3 days (range 2–7 days). Patients exhibited moderate to severe thrombocytopenia with a media nadir platelet count of 48 × 10^9^/L (range 12–87 × 10^9^/L). In addition to reduced platelet counts, three patients were also reported mild hemorrhagic complications (e.g., petechiae, purple) with platelet nadir count ranging from 18 × 10^9^/L to 48 × 10^9^/L. Furthermore, some patients presented with other symptoms, including white blood cell reduction and bone marrow depression.

Interventions in the treatment of thrombocytopenia included PPIs discontinuation, platelet transfusions, administration of steroid and rhIL-11, and other measures. Finally, after PPIs were discontinued in all patients, 11 of 18 patients were successfully recovered (8 with PPI discontinuation alone), and the symptoms of another 7 patients were improved (6 without any treatment). Recovery time was documented in 17 cases, with a median of 4.5 days (range 2–10 days). Additionally, nine patients were switched to alternative drugs for their primary condition (three famotidine, two ranitidine, two omeprazole, one lansoprazole, and one rabeprazole). Rechallenge was reported in three cases, all involving unsuccessful reintroduction of lansoprazole after a previous episode of lansoprazole-induced thrombocytopenia. The average elapsed time from lansoprazole reinitiation to rechallenge was 5 days. Besides, in one esomeprazole-induced case, the patient was switched to dexlansoprazole after improving, but platelet count decreased again. According to the approximate Naranjo scale calculated, 4 cases were classified as “possible” and 14 as “probable.”

## 4 Discussion

Our systematic analysis provides an overview of individual case reports on PPIs-induced thrombocytopenia. We identified 16 case reports involving 18 patients. These cases were sourced from six countries, exhibiting a wide geographic distribution in their reporting locations. However, it is noteworthy that 72.2% of cases (13/18) were reported from the United States and China alone, meaning the resulting evidence may not be universally applicable across all countries. Our findings suggest that PPIs-induced thrombocytopenia can occur in patients of all ages, while the majority of patients were male and concentrated in the age group over 50 years which probably is due to the epidemiological characteristics of acid-dependent gastrointestinal disorders. This may be one of the risk factors for PPIs-induced thrombocytopenia.

By spanning all cases, it was found that nearly every PPI molecule has the potential to cause thrombocytopenia. Rabeprazole has also been documented in this regard but restricted language and full-text, and therefore was not included in the analysis. To date, whether this is the class effect still remains under debate. This study had a few interesting findings. In a lansoprazole-induced case, the patient did not develop thrombocytopenia when they previously took pantoprazole [14]. Similarly, another patient with pantoprazole-induced thrombocytopenia did not experience the same issue when taking omeprazole [24]. Moreover, we also observed that thrombocytopenia did not always recur when patients were switched to other PPIs (e.g., lansoprazole, omeprazole) after discontinuing the culprit drugs [23,26]. These observations suggest that the ADR is most likely an individual drug consequence rather than a class effect.

Among the cases reviewed, pantoprazole was found to cause the highest number of thrombocytopenia cases followed by lansoprazole. A retrospective study revealed that platelet counts of patients were statistically significantly decreased after pantoprazole infusion treatment [28]. On the contrary, Watson et al. screened 385 patients who were prescribed pantoprazole, but no case of pantoprazole-induced thrombocytopenia was observed [23]. Dotan et al. conducted a retrospective cohort study comparing platelet counts between 468 patients prescribed pantoprazole with non-medicated controls, and their results failed to show an increased incidence of thrombocytopenia in the pantrprazole group [29]. Both pantoprazole and lansoprazole belong to first-generation PPIs. Whether the specific type of PPI can be considered as another risk factor for the development of PPIs-induced thrombocytopenia, further evidence is needed to evaluate the association between the type of PPIs and the risk of thrombocytopenia.

Among 18 cases, the median time of platelet counts to start decline is 3 (2–7) days, which is more rapid than what is typically seen in DIT that is usually after 5–7 days. Some studies speculated that it may be related to the prior exposure to PPIs or another molecule with a similar immunologic binding site [15]. Among the cases where thrombocytopenia occurred the very next day, three patients had indeed exposed to PPIs previously [12,21,24], but other cases did not have similar experience [13,15,20,26]. Since DIT is a diagnosis of exclusion and PPI is an uncommon cause of thrombocytopenia, this may lead to misinterpretation or delayed diagnosis. Given that our results indicate thrombocytopenia generally occurs within 7 days after the initiation of PPIs, clinicians should be vigilant for such an event during this critical period.

The primary treatment for thrombocytopenia is withdrawal of PPIs. Other treatments including corticosteroids and platelet transfusion might be also needed depending on the clinical setting. The therapeutic approaches used for thrombocytopenia in the reported cases were consistent with these recommendations, and all patients ultimately achieved recovery or improvement after appropriate and timely management. For patients who still need acid suppression therapy, the alternative option was to switch to other PPI medications or H2 receptor antagonists.

Cases reports and series are often considered relatively low in the level of evidence; however, in three cases, a subsequent recurrence of thrombocytopenia after recalling with the same PPIs provides perhaps the strongest evidence for a causal association. All rechallenges occurred in cases of lansoprazole-associated thrombocytopenia. After recovering 9 days and being re-treated with lansoprazole for upper gastrointestinal bleeding, one patient experienced a greater than 50% reduction in platelet counts within 5 days [14]. Another patient reinitiated lansoprazole therapy for the same reason after a 2-day recovery. Within 3 days, a subsequent decrease in platelet counts from 107 × 10^9^/L to 65 × 10^9^/L was observed [16]. When lansoprazole was restarted for unknown reasons, the platelet count of a third patient dropped from 263 × 10^9^/L to 132 × 10^9^/L within 7 days [17]. In all cases, platelet counts improved rapidly following the discontinuation of lansoprazole.

The mechanisms underlying PPI-induced thrombocytopenia remain unclear. DIT usually occurs through two distinct mechanisms: reducing platelet synthesis by bone marrow megakaryocytes or accelerating platelet destruction by immune- and nonimmune-mediated [30]. In two of the included cases, the reporters speculated that immune-mediated platelet destruction was the most likely mechanism [20,24]. On the contrary, in a case reported by Widyati et al., the mechanism was deemed nonimmune-mediated, as the administration of methyl-prednisolone injection and thrombocyte concentrate transfusion yielded no significant benefit [21]. Additionally, in a case reported by Yu et al., bone marrow suppression was identified as the cause based on positive findings from both bone marrow aspiration and biopsy tests [18].

It is essential to acknowledge the limitations of this study. First, due to the inherent nature of case reports, we cannot draw certainty conclusions or determine incidence or prevalence of such events. Furthermore, the limited number of case reports also affects the reliability of statistical results and restricts the generalizability of findings to broader populations. Second, our search was limited to six databases and we may have missed literature sources not included. At the same time, we did not conduct a specific search for grey literature, introducing an additional source of publication bias. Third, this study protocol was not registered in any database such as PROSPERO. Fourth, detailed information of several cases may be incomplete and Naranjo scores were not reported by the original authors, which prevents the in-depth analysis of the data, hinders appropriate calculation of Naranjo scores, and complicates efforts to better understanding the underlying mechanisms. Last, the included cases originated from diverse clinical setting, with substantial variability in terms of PPIs, patient demographics, and diagnostic tools. Such heterogeneity of the cases precluded data pooling or meaningful comparisons. Future research may focus on collecting comprehensive data by incorporating various types of reports and conducting well-designed large-scale observation studies to document the incidence, risk factors, and possible mechanisms. Furthermore, cytochrome P450 2C19 (CYP2C19) enzyme have been reported to significantly influence the metabolism and exposure level of PPIs. However, whether the CYP2C19 metabolizer status affects the occurrence of PPI-induced thrombocytopenia has not been reported, and none of the cases included in this study mentioned patients’ CYP2C19 genotyping. The mechanisms behind the association of CYP2C19 genotypes and increased risk of PPI-induced thrombocytopenia also require further exploration.

## 5 Conclusion

In summary, PPIs-induced thrombocytopenia is a poorly recognized and rare complication. Given the widespread use of these agents, clinicians should be knowledgeable about and vigilant toward this potential risk. In case of suspicion, immediate drug discontinuation and prompt therapeutic intervention are recommended to ensure clinical safety.

## Supplementary Material

Supplementary Table
