# Supplementary material for: Proton pump inhibitors-induced thrombocytopenia: A systematic literature analysis of case reports
Source: Open Med (Wars). 2025 Oct 29;20(1):20251297. doi: 10.1515/med-2025-1297 (PMC12596856; doi:10.1515/med-2025-1297)
Supplement: Supplementary Table [file med-2025-1297-sm.pdf]

Supplementary material

Table S1: PRISMA statement

Section and topic	Item #	Checklist item	Location where item is reported
TITLE			
Title	1	Identify the report as a systematic review.	title
ABSTRACT			
Abstract	2	See the PRISMA 2020 for Abstracts checklist.	abstract
INTRODUCTION			
Rationale	3	Describe the rationale for the review in the context of existing knowledge.	1
Objectives	4	Provide an explicit statement of the objective(s) or question(s) the review addresses.	1
METHODS			
Eligibility criteria	5	Specify the inclusion and exclusion criteria for the review and how studies were grouped for the syntheses.	2.2
Information sources	6	Specify all databases, registers, websites, organisations, reference lists and other sources searched or consulted to identify studies. Specify the date when each source was last searched or consulted.	2.1
Search strategy	7	Present the full search strategies for all databases, registers and websites, including any filters and limits used.	Supplementary Table S2
Selection process	8	Specify the methods used to decide whether a study met the inclusion criteria of the review, including how many reviewers screened each record and each report retrieved, whether they worked independently, and if applicable, details of automation tools used in the process.	2.2, 2.3
Data collection process	9	Specify the methods used to collect data from reports, including how many reviewers collected data from each report, whether they worked independently, any processes for obtaining or confirming data from study investigators, and if applicable, details of automation tools used in the process.	2.4
Data items	10a	List and define all outcomes for which data were sought. Specify whether all results that were compatible with each outcome domain in each study were sought (e.g. for all measures, time points, analyses), and if not, the methods used to decide which results to collect.	NA

(*Continued*)

Table S1: *Continued*

Section and topic	Item #	Checklist item	Location where item is reported
	10b	List and define all other variables for which data were sought (e.g. participant and intervention characteristics, funding sources). Describe any assumptions made about any missing or unclear information.	NA
Study risk of bias assessment	11	Specify the methods used to assess risk of bias in the included studies, including details of the tool(s) used, how many reviewers assessed each study and whether they worked independently, and if applicable, details of automation tools used in the process.	2.5
Effect measures	12	Specify for each outcome the effect measure(s) (e.g. risk ratio, mean difference) used in the synthesis or presentation of results.	NA
Synthesis methods	13a	Describe the processes used to decide which studies were eligible for each synthesis (e.g. tabulating the study intervention characteristics and comparing against the planned groups for each synthesis (item #5)).	2.3
	13b	Describe any methods required to prepare the data for presentation or synthesis, such as handling of missing summary statistics, or data conversions.	NA
	13c	Describe any methods used to tabulate or visually display results of individual studies and syntheses.	NA
	13d	Describe any methods used to synthesize results and provide a rationale for the choice(s). If meta-analysis was performed, describe the model(s), method(s) to identify the presence and extent of statistical heterogeneity, and software package(s) used.	NA
	13e	Describe any methods used to explore possible causes of heterogeneity among study results (e.g. subgroup analysis, meta-regression).	NA
	13f	Describe any sensitivity analyses conducted to assess robustness of the synthesized results.	NA
Reporting bias assessment	14	Describe any methods used to assess risk of bias due to missing results in a synthesis (arising from reporting biases).	NA
Certainty assessment	15	Describe any methods used to assess certainty (or confidence) in the body of evidence for an outcome.	NA
RESULTS			
Study selection	16a	Describe the results of the search and selection process, from the number of records identified in the search to the number of studies included in the review, ideally using a flow diagram.	3, Figure 1
	16b	Cite studies that might appear to meet the inclusion criteria, but which were excluded, and explain why they were excluded.	NA
Study characteristics	17	Cite each included study and present its characteristics.	Table 1

(*Continued*)

Table S1: *Continued*

Section and topic	Item #	Checklist item	Location where item is reported
Risk of bias in studies	18	Present assessments of risk of bias for each included study.	Supplementary Table S3
Results of individual studies	19	For all outcomes, present, for each study: (a) summary statistics for each group (where appropriate) and (b) an effect estimate and its precision (e.g. confidence/credible interval), ideally using structured tables or plots.	3, Table 2
Results of syntheses	20a	For each synthesis, briefly summarise the characteristics and risk of bias among contributing studies.	Supplementary Table S3
	20b	Present results of all statistical syntheses conducted. If meta-analysis was done, present for each the summary estimate and its precision (e.g. confidence/credible interval) and measures of statistical heterogeneity. If comparing groups, describe the direction of the effect.	Table 2
	20c	Present results of all investigations of possible causes of heterogeneity among study results.	NA
	20d	Present results of all sensitivity analyses conducted to assess the robustness of the synthesized results.	NA
Reporting biases	21	Present assessments of risk of bias due to missing results (arising from reporting biases) for each synthesis assessed.	NA
Certainty of evidence	22	Present assessments of certainty (or confidence) in the body of evidence for each outcome assessed.	NA
DISCUSSION			
Discussion	23a	Provide a general interpretation of the results in the context of other evidence.	4
	23b	Discuss any limitations of the evidence included in the review.	4
	23c	Discuss any limitations of the review processes used.	4
	23d	Discuss implications of the results for practice, policy, and future research.	4, 5
OTHER INFORMATION			
Registration and protocol	24a	Provide registration information for the review, including register name and registration number, or state that the review was not registered.	NA
	24b	Indicate where the review protocol can be accessed, or state that a protocol was not prepared.	NA
	24c	Describe and explain any amendments to information provided at registration or in the protocol.	NA
Support	25	Describe sources of financial or non-financial support for the review, and the role of the funders or sponsors in the review.	Funding information
Competing interests	26	Declare any competing interests of review authors.	Conflict of interest
	27		Data availability statement

(*Continued*)

Table S1: *Continued*

Section and topic	Item #	Checklist item	Location where item is reported
Availability of data, code and other materials		Report which of the following are publicly available and where they can be found: template data collection forms; data extracted from included studies; data used for all analyses; analytic code; any other materials used in the review.	

Table S2: Search strategy employed to identify articles in electronic databases

PubMed-	(Proton pump inhibitor*[tiab] OR omeprazole[tiab] OR esomeprazole[tiab] OR pantoprazole[tiab] OR lansoprazole[tiab] OR rabeprazole[tiab] OR ilaprazole[tiab]) AND (thrombocytopenia[tiab] OR platelet count [tiab])
Web of Science	TS = (proton pump inhibitor* OR omeprazole OR esomeprazole OR pantoprazole OR lansoprazole OR rabeprazole OR ilaprazole) AND (thrombocytopenia OR platelet count)
Scopus-	TITLE-ABS (“proton pump inhibitor*” OR omeprazole OR esomeprazole OR pantoprazole OR lansoprazole OR rabeprazole OR ilaprazole) AND (thrombocytopenia OR “platelet count”)

Table S3: JBI critical appraisal checklist for case reports

Author and reference	Q1	Q2	Q3	Q4	Q5	Q6	Q7	Q8	Overall appraisal	Score out of 8)	Quality
Mukherjee et al. [1]	Y	Y	Y	Y	Y	Y	Y	Y	Include	8	Good
Liu and Han [2]	Y	Y	N	N	Y	Y	N	Y	Include	5	Fair
Liu and Han [2]	Y	Y	N	N	N	N	Y	Y	Include	4	Poor
Saad and Mitwally [3]	Y	Y	Y	Y	Y	Y	N	Y	Include	7	Good
Zlabek and Anderson [4]	Y	Y	Y	Y	Y	Y	Y	Y	Include	8	Good
Deng et al. [5]	Y	N	Y	Y	Y	N	Y	Y	Include	6	Fair
Zheng and Wen [6]	Y	Y	N	N	Y	N	Y	Y	Include	5	Fair
Yu et al. [7]	Y	Y	Y	Y	Y	Y	Y	Y	Include	8	Good
Phan et al. [8]	Y	Y	Y	Y	Y	Y	Y	Y	Include	8	Good
Korkmaz et al. [9]	Y	Y	Y	N	Y	Y	Y	Y	Include	7	Good
Widyati et al. [10]	Y	N	Y	N	Y	Y	Y	Y	Include	6	Fair
Miller et al. [11]	Y	Y	N	N	Y	N	Y	Y	Include	5	Fair
Watson et al. [12]	Y	Y	Y	N	Y	N	Y	Y	Include	6	Fair
Watson et al. [12]	Y	Y	Y	N	Y	N	Y	Y	Include	6	Fair
Kallam et al. [13]	Y	N	N	N	Y	Y	Y	Y	Include	5	Fair
Tas [14]	Y	N	Y	Y	Y	Y	Y	Y	Include	7	Good
Sahad et al. [15]	Y	N	Y	Y	Y	N	Y	Y	Include	6	Fair
Xie et al. [16]	Y	Y	Y	Y	Y	Y	Y	Y	Include	8	Good

Y, yes; N, no; JBI: Joanna Briggs Institute.

Q1: Were patient's demographic characteristics clearly described?.

Q2: Was the patient's history clearly described and presented as a timeline?.

Q3: Was the current clinical condition of the patient on presentation clearly described?.

Q4: Were diagnostic tests or assessment methods and the results clearly described?.

Q5: Was the intervention(s) or treatment procedure(s) clearly described?.

Q6: Was the post-intervention clinical condition clearly described?.

Q7: Were adverse events (harms) or unanticipated events identified and described?.

Q8: Does the case report provide takeaway lessons?.

Quality rating: poor 0–4; fair 5–6; good: 7–8.

References

[1] Mukherjee S, Jana T, Pan JJ. Adverse effects of proton pump inhibitors on platelet count: a case report and review of the literature. Case Rep Gastrointest Med. 2018;4294805.

[2] Liu MC, Han Y. Two cases of thrombocytopenic purpura induced by omeprazole. People's Military Surg. 2015;58(2):190.

[3] Saad M, Mitwally H. Lansoprazole-induced thrombocytopenia in a critically ill patient: a case report. J Pharm Pract. 2020;33(5):700–4.

[4] Zlabek JA, Anderson CG. Lansoprazole-induced thrombocytopenia. Ann Pharmacother. 2002;36(5):809–11.

[5] Deng YH, Xu J, Wang Y. One case of serious thrombocytopenia induced by lansoprazole and meropenem for injection. Chin J New Drugs Clin Rem. 2017;36(1):56–8.

[6] Zheng XM, Wen Y. Thrombocytopenia induced by lansoprazole for injection. Chin J Drug Appl Monit. 2013;10(6):363–4.

[7] Yu Z, Hu J, Hu YJ. Neutropenia and thrombocytopenia induced by proton pump inhibitors: a case report. Drug Saf – Case Rep. 2018;5(1):28.

[8] Phan AT, Tseng AW, Choudhery MW, Makar JB, Nguyen C, Farmand F. Pantoprazole-associated thrombocytopenia: a literature review and case report. Cureus. 2022;14(2):e22326.

[9] Korkmaz U, Alcelik A, Eroglu M, Korkmaz AN, Aktas G. Pantoprazole-induced thrombocytopenia in a patient with upper gastrointestinal bleeding. Blood Coagul Fibrinolysis. 2013;24(3):352–3.

[10] Widyati, Latifah N, Ramadhani M. Pantoprazole-induced thrombocytopenia: unresponsive to corticosteroid and thrombocyte concentrate transfusion. J Pharm Pract. 2023;36(3):711–5.

[11] Miller JL, Gormley AK, Johnson PN. Pantoprazole-induced thrombocytopenia. Indian J Pediatr. 2009;76(12):1278–99.

[12] Watson TD, Stark JE, Vesta KS. Pantoprazole-induced thrombocytopenia. Ann Pharmacother. 2006;40(4):758–61.

[13] Kallam A, Singla A, Silberstein P. Proton pump induced thrombocytopenia: a case report and review of literature. Platelets. 2015;26(6):598–601.

[14] Tas A. Thrombocytopenia as a side effect of pantoprazole. Turk J Gastroenterol. 2013;24(3):295–6.

[15] Sahad, Jeevan RN, David S. A case report on pantoprazole-induced thrombocytopenia. Int J Pharm Sci Rev Res. 2024;84(1):111–2.

[16] Xie MZ, Xu XX, Zeng MF. One case of leukopenia and thrombocytopenia induced by injection of pantoprazole sodium. China Pharmacist. 2017;20(9):1611–53.
